# Brown Spider (*Loxosceles*) Venom Toxins as Potential Biotools for the Development of Novel Therapeutics

**DOI:** 10.3390/toxins11060355

**Published:** 2019-06-19

**Authors:** Daniele Chaves-Moreira, Fernando Hitomi Matsubara, Zelinda Schemczssen-Graeff, Elidiana De Bona, Vanessa Ribeiro Heidemann, Clara Guerra-Duarte, Luiza Helena Gremski, Carlos Chávez-Olórtegui, Andrea Senff-Ribeiro, Olga Meiri Chaim, Raghuvir Krishnaswamy Arni, Silvio Sanches Veiga

**Affiliations:** 1 Departamento de Biologia Celular, Universidade Federal do Paraná (UFPR), Curitiba 81531-970, PR, Brazil; moreirad@pennmedicine.upenn.edu (D.C.-M.); fernando_matsubara@hotmail.com (F.H.M.); zelinda1985@hotmail.com (Z.S.-G.); lidibona@gmail.com (E.D.B.); vane.biomed@gmail.com (V.R.H.); luiza_hg@yahoo.com.br (L.H.G.); senffribeiro@gmail.com (A.S.-R.); olgachaim@gmail.com (O.M.C.); 2 Departamento de Bioquímica e Imunologia, Universidade Federal de Minas Gerais (UFMG), Belo Horizonte 31270-901, MG, Brazil; claragd@gmail.com (C.G.-D.); olortegi@icb.ufmg.br (C.C.-O.); 3 Centro Multiusuário de Inovação Biomolecular, Departamento de Física, Universidade Estadual Paulista (UNESP), São José do Rio Preto 15054-000, SP, Brazil; arni@sjrp.unesp.br

**Keywords:** brown spider, venom, Loxosceles, toxins, biotools, drug targets, novel therapeutics

## Abstract

Brown spider envenomation results in dermonecrosis with gravitational spreading characterized by a marked inflammatory reaction and with lower prevalence of systemic manifestations such as renal failure and hematological disturbances. Several toxins make up the venom of these species, and they are mainly peptides and proteins ranging from 5–40 kDa. The venoms have three major families of toxins: phospholipases-D, astacin-like metalloproteases, and the inhibitor cystine knot (ICK) peptides. Serine proteases, serpins, hyaluronidases, venom allergens, and a translationally controlled tumor protein (TCTP) are also present. Toxins hold essential biological properties that enable interactions with a range of distinct molecular targets. Therefore, the application of toxins as research tools and clinical products motivates repurposing their uses of interest. This review aims to discuss possibilities for brown spider venom toxins as putative models for designing molecules likely for therapeutics based on the status quo of brown spider venoms. Herein, we explore new possibilities for the venom components in the context of their biochemical and biological features, likewise their cellular targets, three-dimensional structures, and mechanisms of action.

## 1. Introduction: Venom Contents and Cellular Targets

Spider venoms are mixtures of biologically active peptides, proteins, glycoproteins, and small organic molecules which interact with cellular and molecular targets to trigger severe, sometimes fatal effects. However, the spider venom could be particularly interesting for the treatment of general diseases as a scaffold for toxin-based drug research. Several venom-based drugs or venom-derived molecules have found extensive use as tools for therapies. For instance, “Captopril”, a competitive inhibitor of angiotensin-converting enzyme, is broadly used and well-established antihypertensive drug developed from a polypeptide toxin isolated from the venom of *Bothrops jararaca*; “Conotoxin” from the sea cone snail *Conus magus* used as an analgesic for severe chronic pain and “exendins”; and, recently, proteins obtained from the saliva of the Gila monster *Heloderma suspectum* benefited the treatment of type II diabetes [1,2,3]. 

Brown spiders (genus *Loxosceles*) has a worldwide distribution of approximately 130 species. Accidents caused by Loxosceles spider envenomation, Loxoscelism, are characterized by dermonecrotic lesions with gravitational spreading, and hence, these accidents are often referred to as necrotic or gangrenous arachnidism. In minor cases, it unveils systemic manifestations, including renal problems and hematological disturbances such as hemolysis, thrombocytopenia, and intravascular coagulation have been observed [4]. Based on proteomic and transcriptomic analysis, the venoms are mainly composed of low molecular mass peptides, proteins, and glycoproteins enriched in molecules in the 5–40 kDa range. There are three classes of “highly expressed molecules” that comprise approximately 95% of the toxin-encoding transcripts in the venom gland [4,5], biochemically characterized as belonging to the family of phospholipases-D, astacin-like proteases and low molecular mass peptides (ICK peptides or Knottins) [5,6,7,8]. Other toxins with low levels of expression have also been identified and include venom allergens, TCTP, hyaluronidases, serine proteases, and serine protease inhibitor [9,10].

Brown spider venom toxins are associated with many cellular changes followed by envenomation, either in humans or in animal-based models for experimental exposure. Rabbit skin exposed to crude venom or human biopsies indicated massive infiltration of inflammatory cells with predominantly polymorphonuclear leukocytes into the dermis, an event associated with dermonecrosis and histologically characterized as aseptic coagulative necrosis [11,12].

Brown spider venom toxins also exhibit indirect and robust activity on blood vessel endothelial cells that cause deregulated activation of leukocytes. The venom activity on endothelial cells was confirmed by experiments performed with umbilical vein endothelial cells, which after venom treatment increased expression and secretion of E-selectin, interleukin-8, and granulocyte macrophage colony-stimulating factor. Additionally, endothelial cells exposed to venom also overexpressed a growth-related oncogene and the monocyte chemoattractant protein-1 [13]. Human keratinocytes treated with venom increased the expression of vascular endothelial growth factor and rabbit aorta endothelial cells treated with venom bound venom toxins on cell surfaces, suffered morphological changes, detached from culture substratum, and their heparan-sulfate proteoglycans were degraded [14,15]. In vivo experiments of animals exposed to venoms indicated endothelial-leukocyte adhesion, transmigration of leukocytes across the blood vessel endothelium and degeneration of blood vessels [15,16]. Other cells targeted by venom toxins are fibroblasts that after exposure deregulated the expression of cytokines genes CXCL1, CXCL2, IL-6, and IL-8 mediators of inflammatory response activation [17]. Erythrocytes represent an additional cell target model of venom toxins. Venom exposure causes hemolysis and morphological changes that seem to depend on a mechanism reflecting a direct effect on cell surface phospholipids such as sphingomyelin and lysophosphatidylcholine leading to the influx of a calcium-mediated pathway by an L-type channel [18,19,20]; and another complement-dependent pathway with the activation of an endogenous metalloprotease, which degrades glycophorins and activates complement and lysis [21].

Platelets are also targets of brown spider venoms. Venom toxin treatment of human platelet-rich plasma induced in vitro platelet aggregation, an event that depends on the generation of lipids derived from platelet membranes [22,23,24]. Additionally, histopathological findings highlighted the marrow depression of megakaryocytes and thrombocytopenia in the peripheral blood after rabbit venom exposure and the generation of thrombus into blood vessels [25,26]. Finally, the cell surface of renal epithelial cells is also targeted by brown spider venom toxins and suffer direct cytotoxicity [27,28].

## 2. Recombinant Toxins: Biotools and Drug Targets

Brown spider venoms are obtained either by electrically stimulating the cephalothorax, which causes the venom glands to extrude the venom, or by gentle compression of the isolated venom glands to produce a venom gland extract. Both methods yield only a few microliters and micrograms of proteins from the venom with similar compositions and biological and biochemical properties [29,30]. Spider lethality following electrical stimulation or in the case of gland extract, the fact that they must be sacrificed, are drawbacks [4,5,31]. Molecular biology techniques for studying brown spider venom toxins have helped to overcome these limitations. At least two venom gland cDNA libraries have been constructed from venom glands of *L. laeta* [32] and *L. intermedia* [27]. Additionally, phospholipase-D family members of different brown spider species have been expressed in bacterial systems [22,23,24,27,32,33,34,35,36]. Site-directed mutant homologs of phospholipase-D have been obtained [18,19,28] and together with recombinant wild-type isoforms have been instrumental for studies on catalysis and determination of the crystal structures of phospholipase-D toxins to provide a better understanding about toxin biology and pharmacology [37,38,39,40,41]. Other recombinant brown spider venom toxins were reported for the astacin-member family [42,43] and an Inhibitor Cystine Knot peptide [44] have also been cloned, expressed and used for studies on the insecticide activities of venoms. A TCTP member-family toxin [10] and a recombinant hyaluronidase from *L. intermedia* venom were heterologously produced and in the case of hyaluronidase used to evaluate its role in dermonecrosis as a spreading factor [6] (see Table 1). 

**Figure 1:**
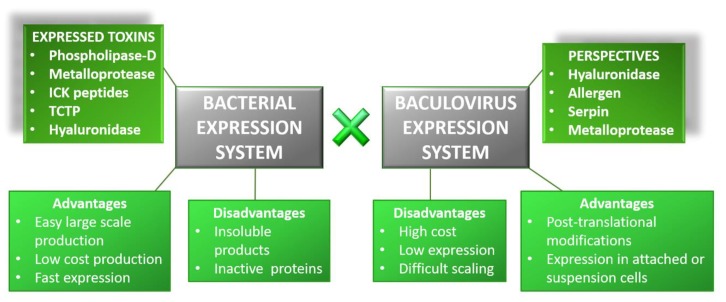
The different systems used for recombinant expression of *Loxosceles* toxins.

Finally, to surpass problems of expressing recombinant molecules using bacterial systems, such as inability to perform post-translational modifications or production of unfolded/insoluble proteins, brown spider recombinant toxins also have been produced using additional expression models as *Spodoptera frugiperda* insect cells [47,48]. The recombinant toxins produced in invertebrate systems might not only be useful for obtaining additional insights into Loxoscelism, but also serve as important tools for future pharmaceutical studies of prospection for drug discovery, serum therapy, and biotechnological applications (see Figure 1).

## 3. ICK Peptides: Analgesic Drug, Neuroprotective Effector and Bioinsecticide

Transcriptome analysis of *Loxosceles intermedia* venom glands revealed that 55.9% of the annotated transcripts encoding toxins are related to ICK peptides, also known as knottins, corresponding to the most representative group of identified toxins in this species [9]. ICK peptides contain the inhibitor cystine knot motif, which is an antiparallel β-sheet structured by a pseudo knot formed by two disulfide bonds and the intervening regions of the peptide backbone that is crossed by a third disulfide bond [49]. The ICK motif provides remarkable thermal, chemical, and biological stability and the peptides are overly stable in human serum for several days, conferring high half-life in gastric fluids and are likely relevant in the development of new drugs and therapies [50,51]. The ICK peptides exert their effects on voltage-gated ion channels expressed in the nervous system of animals [49]. 

**Figure 2:**
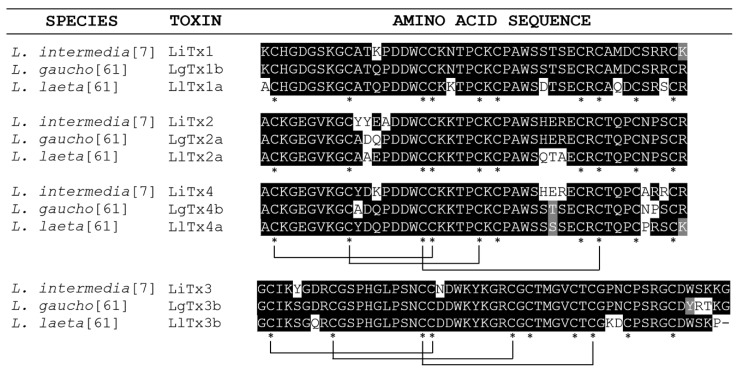
Predicted amino acid sequences of representative sequences of ICK peptides screened in RNA extracts from *L. intermedia*, *L. gaucho* and *L. laeta*. A fraction of *L. intermedia* containing LiTx1, LiTx2 and LiTx3 proved to be toxic against a lepidopteran larva [7]. A LiTx3 orthologue (Li1b) was heterologously expressed and also showed toxic activity in sheep blowflies [64]. Asterisks indicate conserved cysteine residues and the lines below the alignments indicate the pattern of disulphide bonds that form the inhibitor cystine knot motif [64].

For mammals, by acting on these molecular targets, the ICK peptides may be explored for use as analgesics. One example of this potential is the ICK toxin μ-TRTX-Tp1a from the Peruvian green-velvet tarantula *Thrixopelma pruriens* [52]. This toxin is an inhibitor of the Na_v_1.7 sodium voltage-gated channel subtype, which is considered a relevant target for therapeutic solutions related to pathophysiological status such as pain. Recombinant μ-TRTX-Tp1a can revert, in a concentration-dependent manner, spontaneous pain induced in mice by intraplantar co-injection with OD1, a scorpion-venom peptide that is a potent activator of Na_V_1.6 and Na_V_1.7 channels [52,53]. Through in vitro assays, the toxin μ-TRTX-Hd1a, an ICK peptide present in the venom of the spider *Haplopelma doriae*, also activates Na_V_1.7 channels. This toxin, at a concentration of 1 μM, was able to almost completely inhibit Na_V_1.7-mediated currents recorded from oocytes expressing Na_V_ channel subunits [54]. Some authors hypothesized that the molecular targets of the peptide LiTx3 from *L. intermedia* are Na_V_ channels, which were also shown to be the target for the recombinant peptide U2-SCTX-Li1b encountered in *L. intermedia* venom [7,44]. Na_V_ or Ca_V_ channels may be the targets of the peptides LiTx1 and LiTx2 (see Figure 2). Different authors identified and sequenced several peptides that belong to the LiTx family encoded in the venom gland of *L. intermedia* [9]. Thus, the *L. intermedia* venom contains an impressive arsenal of molecules potentially important as an analgesic against acute and chronic pain conditions [9]. In a recent review, Netirojjanakil and Miranda [55] affirm that the challenge of venom-derived peptide therapeutic development remains in improving selectivity to the target and in the delivery of these peptides to the sites of action in the nervous system.

Other putative targets for spider ICK peptides are the acid-sensing ion channels (ASIC) encountered in the human central and peripheral nervous system, mainly in neurons [56]. These channels are specifically pH-modulated (low pH) in the extracellular environment. Depending on the decreasing pH that the neuronal cells are exposed to, the channel can be activated, which initiates sodium transport. The ASIC1a, a subunit of some ASIC channels, has been reported as a target that can be modulated, thus influencing pain or stroke clinical conditions [50]. The ICK peptide PcTx1 from the tarantula spider *Psalmopoeus cambridgei,* a known ASIC1a specific blocker, is a potent analgesic when intrathecal and intracerebroventricular injected in mice, producing effects similar to morphine [57]. 

Furthermore, PcTx1 toxin was neuroprotective in focal ischemia studies conducted in adult mouse: intracerebroventricular administration of this ICK peptide was able to reduce the percentage of the ipsilateral hemisphere infarct by more than 50% after 1 h of transient middle cerebral artery occlusion [58]. The authors also demonstrated the PcTx1 neuroprotective effects when this peptide was intracerebroventricular-injected in newborn piglets subjected to hypoxia-ischemia [59]. The ICK structural motif confers increased resistance to proteolysis, unlike the linear peptides that are highly susceptible to this process. Thus, ICK peptides and their respective biological properties possess significant potential for use in basic and applied research. The identification and characterization of these peptides in venoms from *Loxosceles* spiders may lead to the development of important appliances for the therapy of diseases affecting millions of people worldwide. 

Studies of ICK peptides PnTx2-6 and PnTx2-5 identified in the venom of *Phoneutria nigreventer* spider shows that these toxins may be applied in the treatment of erectile dysfunction [60]. PnTx2-6 induces priapism in mice even after cavernosal denervation and increases relaxation in rat cavernous strips and in vivo, but induces significant side effects [61]. These unwanted effects and the difficulty to obtain this peptide in large amounts led to the design of a smaller peptide based on PnTx2-6 sequence, which showed promising features for erectile dysfunction treatment [62].

Another application for ICK peptides is the use as bioinsecticides. These peptides act upon targets in the peripheral or central nervous system of the insect inducing paralysis or lethality. The target may be sodium or calcium voltage-dependent channels, as well as calcium-activated potassium channels, presynaptic nerve terminals, or N-methyl-D-aspartate (NMDA) receptors [63]. Concerning to peptides from *L. intermedia*, after chromatography steps, the fraction containing LiTx1, LiTx2, and LiTx3 peptides proved to be toxic for the lepidopteran larvae of *S. frugiperda* resulting in flaccid paralysis or even death [7]. By studying *Loxosceles intermedia* venom gland, a cDNA of a 53 amino acid ICK peptide was obtained, heterologously expressed in the periplasm of *Escherichia coli*, and after purification caused irreversible flaccid paralysis in sheep blowflies. Such ICK peptide is biologically conserved in two other species: *L. laeta* and *L. gaucho* [64]. This biological activity of ICK peptides has an important biotechnological significance since it can lead to the development of effective bioinsecticides against pests of economic interest.

## 4. Phospholipase-D: Treatment of Loxoscelism, Chemotherapy Drugs, and Anti-Inflammatory Drugs

The phospholipase-D (PLD) family of toxins, also known as dermonecrotic toxins, are the most studied and well-characterized components in *Loxosceles* venoms. These molecules catalyze the degradation of cell membrane phospholipids, such as sphingomyelin (SM), at a terminal phosphodiester bond to release choline and produce ceramide 1-phosphate (C1P) [65]. Also, PLDs can degrade lysophosphatidylcholine (LPC) in an Mg+2 dependent-manner and release lysophosphatidic acid (LPA) [66]. The PLD toxins also catalyze transphosphatidylation of the phosphodiester linkage between the phosphate and head groups of some phospholipids, forming alcohol, and a cyclic ceramide phosphate (CCP) when using SM or cyclic phosphatidic acid (CPA) when using LPC [44]. More than 20% of the transcripts from the *L. intermedia* venom glands corresponded to PLDs [9]. Moreover, the *L. intermedia* transcriptome analysis criteria for bioinformatics screening provided new putative isoforms of PLD, which can be included as new groups in the LoxTox family [67]. These data corroborate the identification of eleven PLD isoforms in the venom of *L. gaucho* and at least twenty-five in the *L. intermedia* venom [68,69]. The PLDs are responsible for a large variety of disturbances in Loxoscelism. Both native and recombinant forms of PLDs have been reported to trigger dermonecrotic lesions, cause an increase in vascular permeability, produce an intense inflammatory response at the inoculation site and at a systemic level, promote platelet aggregation, hemolysis, nephrotoxicity, and even lethality in controlled experiments [18,19,22,28]. 

**Figure 3:**
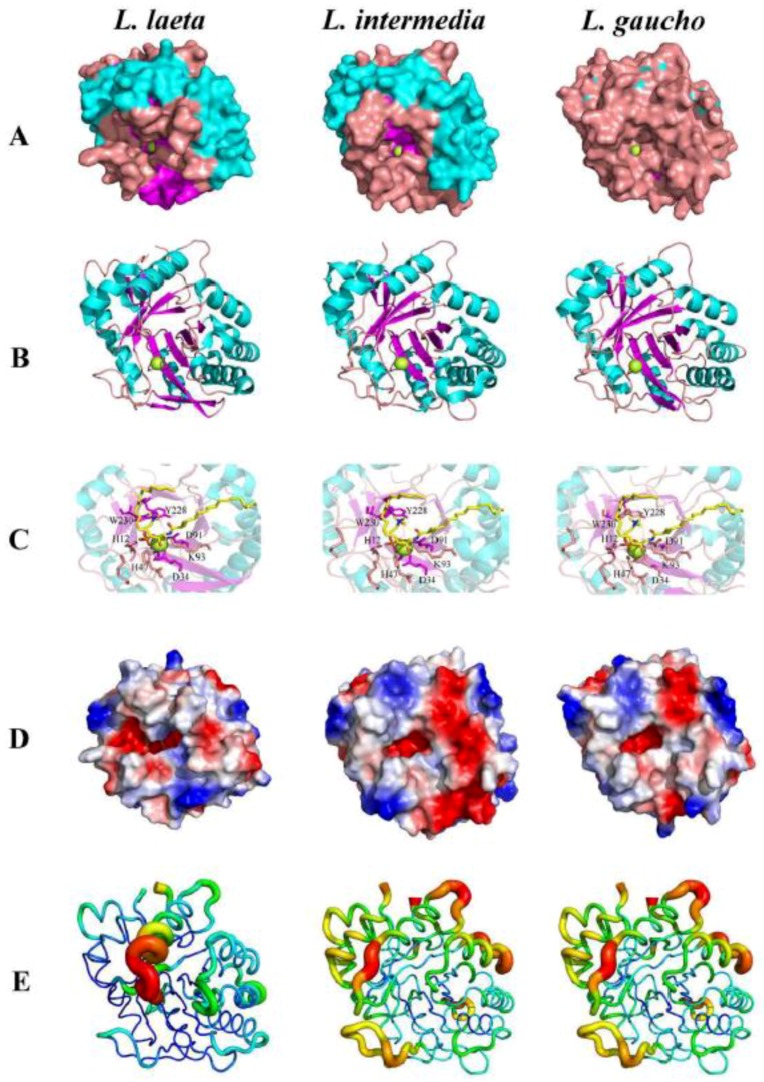
Structural comparison of venom phospholipase-D from *Loxosceles gaucho*, *Loxosceles intermedia* and *Loxosceles laeta*. (**A**) Surface view and (**B**) Ribbon view showing regions of α-helix (cyan), β-Sheet (magenta), Loop (salmon) and Magnesium ion (green sphere). (**C**) Zoom view of the catalytic site showing conserved amino acid residues (H12, H47, E32, D34, K93, Y228 and W230), Magnesium ion (green sphere), and the sphingomyelin (yellow stick). (**D**) Electrostatic surface colored by charge, from red (-2 kV) to blue (+2 kV). (**E**) Flexibility representation generated by b-factor putty, from more rigid regions in blue and green to more flexible regions yellow and red. Models are generated according to PDB codes: 1XX1 (*Loxosceles laeta*) and 3RLH (*Loxosceles intermedia*). Model for *Loxosceles gaucho* was generated using Modeller Program with the LgRec1 sequence from GenBank code: JX866729. PyMOL originated in all figures.

The crystal structures of phospholipases-D from *L. laeta*, *L. intermedia, and L. gaucho* brought to light several insights about the overall conformation and catalytic mechanism and will direct the inhibitor design [37,38,39,40]. The single chain PLDs fold to form a distorted (α/β)8 barrel where the inner barrel surface lines with eight parallel β-strands linked by short flexible loops to eight α-helices that form the outer surface of the barrel (Figure 3). The catalytic loop is stabilized by a disulfide bridge (Cys51 and Cys57) in phospholipases-D class I (*L. laeta*) and a second disulfide bridge (Cys53 and Cys201) is present only in the phospholipases-D class II (*L. intermedia*), which links the catalytic loop to the flexible loop to significantly reduce the flexibility of the latter loop, as evidenced by the mean temperature factors [38,39,40]. Because the α-helices, β-strands, and loops vary in length and character, the barrel is significantly distorted. The interior of the barrel is densely packed with hydrophobic amino acids, and the short N-terminal section and the C-terminal extension, which contains a short α-helix, a β-strand, and a random coiled region serve to cap the torus of the far side of the barrel. The surface loops forming the near side of the barrel are mainly hydrophobic, and a narrow cavity provides access to the catalytic site, which is characterized by a ring of negatively charged amino acids [38,39,40]. This ring is considered to be the choline-binding site that interacts with Tyr228. The catalytic and Mg2+ binding sites are located in a shallow depression and contain His12, Glu32, Asp34, Asp91, His47, Asp52, Trp230, Asp233, Tyr228, and Asn252, which are fully conserved in *Loxosceles* PLDs. Recent site-directed mutagenesis studies of PLDs indicated the involvement of two histidines that are close to the metal ion-binding site in the acid-base catalytic mechanism. Based on the structural results, His12 and His47 of PLD have been identified as the key residues for catalysis and are assisted by a hydrogen bond network that involves Asp52, Asn252, and Asp233. The metal ion is coordinated by Glu32, Asp34, Asp91, and solvent molecules. The substrate is stabilized by Tyr228, and Lys93 [70]. 

In order to achieve an inhibitor prototype, we searched for possible inhibitors of the PLD from *L. intermedia,* and we discovered that halopemide derivatives could bind and inhibit the recombinant toxin [71]. These molecules, developed to target human PLD and treat cancer, could serve as a prototype for the design of new molecules to specifically treat Loxoscelism [72]. PLDs inhibitors exhibit anti-inflammatory activity and significantly reduce oxidative burst, leukocyte migration, degranulation, and inflammatory cytokine production [73]. 

Another interesting industrial application of the brown spider recombinant PLD is in the production of diverse beneficial endogenous bioactive lipids such as palmitoylethanolamide (PEA) that have anti-inflammatory and anti-neurodegenerative properties [74]. PEA was shown to reduce tumor necrosis factor alpha, pro-inflammatory cytokines, and prostaglandin E2 in the plasma. The neuroprotective effects of PEA are in part the result of its effects on downregulating the inflammatory cascade. Indeed, many neurodegenerative diseases are associated with a strong inflammatory component, such as Alzheimer’s disease, Parkinson’s disease or multiple sclerosis.

## 5. Proteases: Matrix Modulator and Thrombolytic Agent

Proteolysis on both the extracellular matrix (ECM) and cell surface modulates symptoms of envenomation by *Loxosceles* spiders [9,75,76]. Studies have revealed the proteolytic activity of the venom obtained directly from gland extracts of *L. rufescens and L. intermedia* (thus excluding potential contaminants with oral egesta) [30,77]. Also, transcriptome analyses from venom glands have encountered toxin-coding transcripts for serine-proteases and metalloproteases [9,78]. Proteases were considered highly expressed toxins in the venom glands, reaching about 23% in *L. intermedia*. Notably, metalloproteases were already reported in the whole venom of several species of *Loxosceles* genus [29,42]. 

Metalloproteases of *Loxosceles* venom are zinc-dependent endopeptidases that hydrolyze a variety of ECM molecules. For instance, two metalloproteases were characterized in the *L. intermedia* venom: Loxolysin A (20-28 kDa) and Loxolysin B (32–35 kDa), which hydrolyzes fibronectin, fibrinogen and denatured type I collagen [29]. Moreover, cDNA library of *L. intermedia* venom gland showed metalloprotease toxin-related sequences identified as astacin-like proteases due to the presence of enzymatic catalytic domain and structural motifs (HEXXHXXGFXHE “catalytic domain” and MXY “methionine-turn”). The recombinant form of LALP was able to induce morphological changes, such as loss of adhesion of muscular aorta cells in vitro, and hydrolyzed purified fibrinogen and fibronectin [29,30]. Furthermore, the relevance and conservation of metalloproteases for *Loxosceles* genus was also reported as a gene family by the identification of other four different LALP isoforms in the gland venoms of three *Loxosceles* species [43]. Recently, the whole venom complexity of the same species was analyzed using subproteomic and proteomic approaches for LALPs [8]. The majority of LALP-related molecules showed basic or neutral isoelectric points ranging between 24kDa and 29kDa for the three *Loxosceles* venoms. 

Nevertheless, the venom toxins revealed different patterns of proteolytic activity upon gelatin and fibrinogen as substrates. These findings corroborate to the existence of a larger group of LALPs and propose the idea of a more intricate role for *Loxosceles* metalloproteases than previously suggested in Loxoscelism [41]. 

The enzymatic activities of brown spider proteases upon ECM, cell surface, and different proteins highlight these molecules as useful biotools [32]. Considering the physiopathological events related to ECM degradation, these proteases could be used for the establishment of protocol in pharmaceutical research for example as matrix modulator in healing processes, mostly in wound debridement removing the dead or damaged tissue. Theses proteases can also assist in antibiotic therapy, once some pathogenic bacteria produce biofilms, which in some cases help bacterial adhesion to host tissue and makes the penetration of the antibiotics administered difficult. Another possible application of brown spider proteases are as thrombolytic agents, due to their fibrinolytic activity and anticoagulant property. Although, further studies are necessary to investigate their potential as a therapeutic agent for thrombosis. Besides that, these proteases could participate as biotools in cleaner solutions for denture and contact lenses by removing impregnated proteinaceous materials and prolonging their viability [31,79,80].

## 6. Serine Protease Inhibitors: Anti-Proliferative and Anti-Metastatic Activities, Adjuvants in the Proteolytic Inhibition and Agricultural Pest Regulators

Inhibitors of serine proteases are grouped in three superfamilies according to their mechanism of action: canonical inhibitors, non-canonical inhibitors and serpins [81,82]. More than 1500 serpin sequences have been identified in the genomes of living organisms, and although they are serine protease inhibitors, several have additional functions as inhibitors of members of the cysteine protease family, caspase, and cathepsin [83,84]. Rather than being considered promiscuous, they appear selective in the sense that the targeted enzymes are often part of a conserved biological mechanism [85]. Human serpins are the most well-characterized serine protease inhibitors because they have a central role in several physiologic processes such as coagulation, fibrinolysis, development, malignancy, inflammation, and fertilization. Serpins inhibit a variety of circulating proteases as well as proteases that are activated or released in tissue, the reason why these molecules are involved in different pathologies and dysfunctions [86,87,88]. For instance, serpinopathies can occur due to genetic mutations that lead to inactivation of serpins by protein aggregation with loss of function [89]. 

Mammal serpins and serpin-derived peptides have been used in preclinical tests as a treatment option for serpinopathies, such as alpha-1 antitrypsin deficiency with emphysema, inflammatory vascular diseases from transplant, inflammatory vasculitic syndromes, and even sepsis with disseminated intravascular coagulation. Serpin-derived peptides are also in development as a new approach to block the adverse effects of serpins upregulated in cancer or inflammation [89].

Serpins are present in animal venoms from snakes, snails, sea anemones, wasps, scorpions, spiders and in ticks’ saliva [9,90,91]. The transcriptome analyses of venom glands of *Loxosceles* genus spiders revealed transcripts encoding serine protease inhibitors. The cDNA library of the *L. intermedia* venom gland showed the presence of one serine protease inhibitor-related transcript [9]. In the *L. laeta* transcriptome, 0.6% of the transcripts were related to this same function [78]. The reasons for the presence of protease inhibitors in animal venoms and the physiological targets of these molecules are poorly understood. It has been proposed that these serine protease inhibitors would protect venom toxins from the protease actions of the prey body and manipulate host defenses [92,93]. These inhibitor sequences found in *L. intermedia* and *L. laeta* transcriptome analyses are similar to some tick and mammal serpins. *L. intermedia* serpin shows higher similarity to tick saliva serpins, such as from *Amblyomma americanum*, which is also an arachnid. It is known that arthropod serpins mediate several hemostatic and anti-inflammatory effects in mammalian blood [94]. The saliva serpin 6 and 19 from *A. americanum* contribute to hemostasis dysregulation of the host, facilitating blood feeding. At high molecular excess, recombinant Serpin 19 inhibits several serine proteinases of the blood-clotting cascade and forms inhibitory complexes with Factor Xa, Xia, and trypsin [95,96]. The best-characterized tick saliva serpin is the *Ixodes ricinus* immunosuppressor, Iris, which acts as an anticoagulant and inhibits the secretion of pro-inflammatory cytokines [97]. 

Inflammation-related complications are already focus of studies using serpins with therapeutic potential: C1 Esterase Inhibitor (C1NH) has been investigated toward many inflammation-related complications, and experimental treatment with this serpin showed decreased tissue complement activation and attenuated renal, intestinal and lung injury in a porcine model for hemorrhage [98]. Recombinant Alpha 1 antitrypsin-Pittsburgh has been investigated as a therapy for sepsis, attenuating the characteristic decreases in the functional concentrations of antithrombin, FXI, and fibrinogen [99].

Another putative biotechnological application for the serine protease inhibitors from *Loxosceles* venoms could be in the oncology field as antitumoral drugs focusing on the prevention of invasion and metastasis. A particular member of the serpin family, maspin, has been previously studied because of its inhibitory potential in breast and prostate cancer development [100]. Recently, the designing novel maspin-based chemotherapeutic agents with improved anti-cancer potency was suggested [101]. Katsukawa and co-workers [102] suggested that serpin 5 secreted by epithelial cells acts as a component of the extracellular surveillance system that facilitates the clearance of premalignant epithelial cells. Scabies mite serine protease inhibitors (SMSs) of the serpin superfamily were reported to interfere with all three pathways of the human complement system at different stages of their activation [103].

Another use of protease inhibitors from the *Loxosceles* spider venom could be the insertion of genes encoding serpins in cultivars, as previously described for some serine protease inhibitors applied in alfalfa, potato, cotton, and tobacco crops, which significantly incremented their resistance to attack by insects and predators [104]. 

Overall, the advantages of exploring the biotechnological potentials of serpins benefit from modulating serine protease activity by the introduction of serpin, as a therapeutic, or the blockade of serpins (serpin inhibitors). Several drugs currently in use or in development aim to replace dysfunctional serpins and to block adverse effects induced by aberrant protease or serpin actions [89].

## 7. Hyaluronidases: Adjuvant for Drug Absorption, Diagnostic Allergy Tests, Delivery of Chemotherapy and Contraceptive Molecules

Hyaluronidases (HAases) were reported in *L. deserta*, *L. rufescens*, *L. gaucho*, *L. intermedia*, *L. laeta* and *L. reclusa* venom. Proteomic and transcriptomic studies corroborated these data [9,78,105]. *L. intermedia* HAases were described as endo-β-*N*-acetyl-D-hexosaminidases. The first and unique *Loxosceles* HAase produced in a recombinant form is the Dietrich’s HAase from *L. intermedia*, which presents 45 kDa and in vitro activity on hyaluronic acid (HA) and chondroitin sulfate (CS) degradation [6]. This study using the recombinant isoform confirmed the participation of the HAase as a spreading factor of the dermonecrotic lesion and the inoculated venom. A conserved cystinyl scaffold in the venom HAases suggests a structural similarity with other HAases. Mapping of the sequences of venom HAases on the crystal structure of *Apis mellifera* HAase supported this. Many venom HAases have been found to share a sequence homology of about 36% with that of spermatozoan PH-20, the testicular HAase that participates in fertilization [106]. In contrast to mammalian and microbial HAases, which are extensively studied for their physiological significances, the venom enzymes have received less attention [107]. 

Enzymes such as HAases have several biomedical applications by cleaving HA in tissues, they render tissues more permeable to injected fluids (spreading effect), increase membrane permeability, and reduce viscosity [108]. HAases have been used to reduce the extent of tissue damage following extravasation of parental infusions as electrolytes, chemotherapeutic agents, and antibiotics. HAases can be used therapeutically to promote resorption of excess fluids, to increase the effectiveness of local anesthesia. Some clinical studies reported that HAases could increase the speed of absorption of other substances, such as a human HAase (rHuPH20) increased insulin dispersion and accelerated its absorption [108]. 

Venom HAase of arthropods is a major allergen that can induce severe and occasionally fatal systemic IgE-mediated anaphylactic reactions in humans [106]. Determination of structural moieties responsible for the observed allergic potency will have great importance in clinical implications. Recently, the immunogenic potential of the HAase recombinant protein from social wasp (Vespidae) was suggested to its use for developing a diagnostic allergy test, as well as for specific immunotherapy [106,108]. 

The inhibition of the hydrolytic activities of HAases is also a very promising biotechnological output. Inhibitors of HAase are potent regulating-agents, which play a role in the maintenance of balance between the anabolism and catabolism of HA. The brown spider HAase action during the envenomation contributes to the local effects of the whole venom on the skin as well as potentiating systemic endeavors [106]. Antiserum and inhibitors for the spider HAase are potential agents to attenuate both local and systemic effects of Loxoscelism, as it was already demonstrated for other venom HAase [107]. The blockage of HAase activity would widen the time gap between the bite and the antivenom administration by limiting the diffusion of venom components, and it would reduce the antivenom load to achieve effective neutralization and, therefore, to reduce the collateral effects of the serum therapy [107]. 

## 8. TCTP: Antiparasitic Effect, Dental Restoration and Drug Delivery

The translationally controlled tumor protein (TCTP), also known as histamine-releasing factor (HRF), is a highly conserved, ubiquitous protein that has both intracellular and extracellular functions [109]. TCTP promotes allergic response in mammalian tissues by inducing the release of histamine from basophiles or mast cells. TCTP family proteins have already been described in the gland secretion of other arthropods, as ixodid ticks [110]. In *Loxosceles* venoms, it was encountered in *L. intermedia* and *L. laeta* transcriptome studies, and immunological cross-reaction studies suggest the presence of this toxin in *L. gaucho* venom [111]. Recombinant *L. intermedia* TCTP (LiRecTCTP) causes edema, increases vascular permeability, and is related to the inflammatory activity of the venom [10]. A TCTP protein was described in tarantula *Grammostola rosea* venom gland [112] and *Scytodes* spiders [113]. 

It is suggested that TCTP may play a crucial role in the establishment, maintenance, and pathogenesis of parasite infections. In a mice trial evaluating *Plasmodium* TCTP (42.2% of similarity with LiRecTCTP using EMBOSS Needle tool) as a malaria vaccine, a significant reduction of parasitemia in the early stages of the infection was seen [114,115]. TCTP from worms were also suggested as a putative filarial protein for diagnostic purposes [116]. In *Plasmodium*, TCTP has been shown to bind directly the anti-malarial drug artemisinin and to have higher expression levels on increased drug resistance conditions [117]. 

Furthermore, TCTP was detected in the biological fluid of asthmatic and parasitized patients [109]. Human TCTP (54.9% of similarity with LiRecTCTP using EMBOSS Needle tool) was described as a therapeutic target in asthma and allergy [109,118]. The N-terminal residues of TCTP (residues 1-10, MIIYRDLISH) form a protein transduction domain (PTD); these domains are recognized as promising vehicles for the delivery of macromolecular drugs. Different studies had already pointed out the TCTP PTD and some derivatives as efficient vehicle for drug delivery [119,120]. Recombinant TCTP from the prawn *Penaeus merguiensis* (57.2% of similarity with LiRecTCTP using EMBOSS Needle tool) has been studied as a supplement in dental restorative materials [121]. Recently, this TCTP was shown to promote osteoblast cells proliferation and differentiation, which improve restoration materials and their properties of inducing bone cell proliferation [114]. 

TCTP is described as a multifunctional protein involved in several cellular processes and is highly conserved [122]. In summary, LiTCTP is a promising toxin and potential target model with regards to its broad biotechnological applications for general biology fields (toxinology, parasitology, allergy, and oncology), and biomaterial research (dental restoration and drug delivery).

## 9. Brown Spider Venom Toxins: New Immunotherapies

Spider envenoming treatment by antivenom injection is controversial and no adequate clinical trials have been conducted so far. Besides, the best treatment protocol, which involves a combination of serum therapy and other drugs, remains to be established. Nonetheless, this treatment has the best therapeutic potential for treating loxoscelism as it has been shown to be efficient when initiated in time. In 2009, it was demonstrated that the equine-derived polyvalent loxoscelic antivenom produced against an equal proportion mixture of *L. intermedia, L. gaucho* and *L. laeta* venoms was efficient in reducing the envenoming effects of twice the minimum necrotizing dose of *L. intermedia* venom when administered to rabbits up until 12 h after venom injection [123]. Loxoscelic antivenom was also able to reduce the necrotic area caused by the venom even 48 h post-envenoming [123]. 

Traditional antivenom production is highly inefficient since it is difficult to obtain the necessary amount of venom to perform the immunization program. Each spider produces only a few microliters of venom. Taking Brazil as an example: there is a demand for 22,000 ampoules of antiloxoscelic serum per year. To produce this amount, 1,800 mg of venom is needed and requires venom extraction from approximately 36,000 spiders. Another important consideration is the toxic effect that crude venom exerts in the producer animal, commonly leading to the development of ulcers and abscesses, compromising the animal’s health [124]. 

The availability of such a diverse group of recombinant PLDs represents an alternative source of immunogens for antivenom production. Recombinant PLDs showed to be active and was used to immunize mice, leading to the production of cross-reactive antibodies, which were also able to protect the animals against lethal effects of the whole venom [124]. In addition, horses immunized with this recombinant PLD developed antibodies that were able to cross-react and neutralize the lethal and dermonecrotic effects of *L. laeta* whole venom from Peru [125]. Antivenom produced using a combination of recombinant PLDs from *L. laeta* and *L. intermedia* as immunogens was tested. The antibodies showed cross-reactivity with the three main species of *Loxosceles* in Brazil and could neutralize their toxics effects more efficiently than an anti-arachnidic commercial antivenom, produced using a combination of *L. gaucho, Phoneutria nigriventer,* and *Tityus serrulatus* venom [126]. A combinational approach was also used with recombinant PLDs from *L. reclusa* and *L. bonetti* spiders (from North American origin), and also *L. laeta*, to immunize horses [127]. Individual immunization was also performed in rabbits. The monovalent antivenoms showed no cross-neutralization between North and South American species. The F(ab)2-based horse polyvalent antivenom showed high neutralizing potency, indicating a possible path for the development of a pan-american anti-loxoscelic antivenom. From *L. gaucho,* only one recombinant PLD has been produced, named LgRec1, which was also able to elicit neutralizing antibodies against the toxic effects of the analogous crude venom [46]. 

Other approaches for improving production of antiloxoscelic antivenom include the use of synthetic peptides mimicking mapped epitopes within the sequence of the main toxins from these venoms. The authors mapped epitopes from rLiD1 by SPOT assay using anti-rLiD1 antibodies [124]. Six regions were mapped, and the corresponding peptides were synthesized and used to develop an immunization protocol in combination with rLiD1, in different proportions. Although a mixture of six peptides was used, one of them appeared to be immunodominant. All of the used proportions between recombinant PLD and peptides were able to elicit antibodies that recognized rLiD1 by ELISA and neutralized the noxious effects of rLiD1 both in vitro and in vivo. Neutralizing antirLiD1 antibodies mapped an immunodominant epitope in a SPOT assay [124]. This epitope has 27 residues and is related to the active site of the enzyme. When used as an immunogen, the 27-mer peptide induces protective antibodies in mice and rabbit, protecting them from lethal and dermonecrotic effects of the venom respectively [128]. 

A neutralizing monoclonal antibody against *L. intermedia* was produced and its corresponding epitope was mapped by Phage Display techniques [129]. Synthetic peptides corresponding to the mapped epitopes were used as immunogens in rabbits, and the elicited antibodies were able to neutralize 60% of the dermonecrotic activity and 80% of the hemorrhage caused by *L. intermedia* crude venom [130]. To improve the use of molecules mimicking epitopes, chimeric recombinant proteins combining mapped epitopes in the same molecule was conducted with promising results. This approach was used to express in *E. coli* a chimeric non-toxic protein containing three rLiD1epitopes. These epitopes were previously identified, characterized and validated as potentially neutralizing regions [128,129,130]. The immunogenicity of the chimeric protein was assessed in rabbits and the developed antibodies neutralized the toxic effects of rLiD1 [131]. The chimeric protein was used in an immunization protocol either alone or combined with crude venom (a mixture of *L. intermedia, L. gaucho* and *L. laeta*). The three initial doses of the immunization schedule were composed of the venom, and the subsequent dose was composed of the chimeric protein. Compared to the traditional production of antivenom, this combined protocol was able to induce a similar ELISA and Western Blot serum reactivity towards the venoms of the three *Loxosceles* species. In neutralization assays, the sera produced by immunization with the combination of crude venom and chimeric protein met the necessary potency requirements for the actual production of antivenom for therapeutic use in humans. Although the immunization protocol using only the chimeric protein was not completely successful, the combined protocol used 67% less venom when compared to the traditional one, which is significant [132]. 

Finally, a multi-epitopic chimera containing linear and conformational sequences of the phospholipase-D (dermonecrotic) toxins from the *Loxosceles intermedia* and *L. laeta* venoms, hyaluronidases, and astacins (spreading factors), from the *L. intermedia* venom was produced and used as antigen to generate a polyvalent serum in rabbits. This serum was able to recognize the venoms of three different species of *Loxosceles* involved in accidents in South America (*L. intermedia*, *L. gaucho,* and *L. laeta*), besides neutralizing in vitro the enzymatic effects of crude venoms and lethality and dermonecrotic activities in vivo [133]. These results open the possibility of using this synthetic antigen as a tool to obtain new antivenom sera or as antigens for the production of anti-*Loxosceles* vaccines.

## 10. Conclusions

For most people, spiders often evoke fear and repulsion; however, their venoms form an extensive repertoire of novel molecules, which result in a wide range of biological processes. Several molecules have therapeutic and biotechnological potential and serve as the motif for the development of new molecules with industrial and medicinal applications (Table 2). 

**Figure 4:**
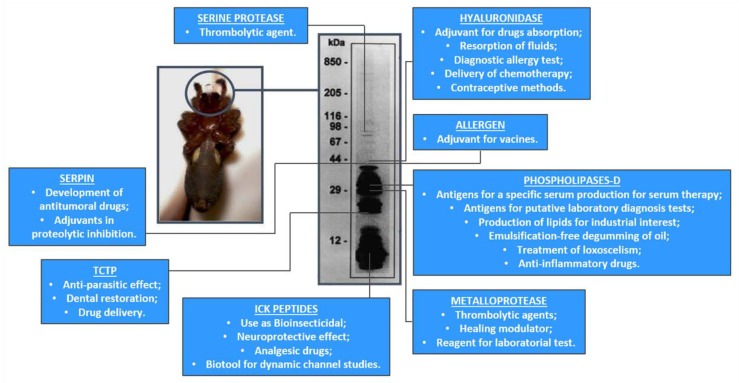
A summary of the *Loxosceles* spider venom toxins and their potential biotechnological and pharmacological applications.

After analyses of the brown spider venom glands transcriptome, an expansion of the number of biotechnologically and pharmacologically relevant molecules and drug targets occurred and inspired the identification of a significant number of potential drug targets that can lead to the synthesis of new inhibitors. Moreover, some of these toxins could be used by themselves, as biotools in biological science projects, pharmaceutical industry or serum therapy, corroborating the high impact of the brown spider venom molecules for biotechnological development. Recent advances in the expression of recombinant toxins have overcome the necessity of obtaining them from a large number of spiders, as they have a very tiny amount of venom (Figure 4). Phospholipases-D, Astacin-like metalloproteases, Inhibitor Cystine Knot peptides, Hyaluronidase and TCTP have been successfully expressed in prokaryote system. The current goal is to promote their biochemical, biological and structural characterization for use them as models for novel therapeutics. All these potential uses and applications for the brown spider toxins are very encouraging. This could appear somewhat remote from the goal of treating diseases, but the challenge of studying the pharmacological properties of the toxins contained in the brown spider venoms can be very surprising. Once these toxins are prove to be versatile, they could be the missing key to discovery an important new drug for a major sickness.

## Figures and Tables

**Table 1:** Characteristics of recombinant toxins of *Loxosceles* spider venoms.

Toxin Family	MM (kDa)	Species	Biological Characteristics	N° of Sequences	PDB
PLD	30–35	*L. arizonica* [45] *L. boneti* [36] *L. gaucho* [46] *L. intermedia* [28] *L. laeta* [32] *L. reclusa* [34] *L. similis* [35]	-Hydrolysis of phospholipids; -Transphosphatidylation; -Dermonecrosis; -Inflammatory response; -Lethality; -Hemolysis; -Platelet aggregation; -Edema; -Nephrotoxicity; -Cytotoxicity; -Cytokine activation; -Complement activation.	199	1XX1 2F9R 3RLH 3RLG 4RW5 4RW3
Metalloprotease	30	*L. intermedia* [42]	-Hydrolysis of Gelatin, Fibronectin and Fibrinogen; -Cytotoxicity.	3	N.A.
ICK peptides	12	*L. intermedia* [44]	-Insecticidal activity.	1	N.A.
Hyaluronidase	45	*L. intermedia* [6]	-Hydrolysis of hyaluronic acid and chondroitin sulfate; -Dermonecrosis spreading.	1	N.A.
TCTP	22	*L. intermedia* [10]	-Edema; -Vascular permeability.	1	N.A.

N.A: not applied.

**Table 2:** Overview of potential biotechnological and pharmacological applications of toxins from *Loxosceles* spider venoms.

Toxin Family	Potential Uses as Biotools	Potential Uses for Drugs Design
Phospholipase-D [22,23,24,27,33,69,105]	-Antigens for a specific serum production for serum therapy; -Antigens for putative laboratory diagnosis tests; -Production of lipids for industrial interest; -Emulsification-free degumming of oil;	-Treatment of Loxoscelism; -Anti-inflammatory drugs, -Neuroprotective drugs; -Adjuvant drugs for cancer chemotherapy;
Metalloprotease [41,42,43]	-Trombolytic agents	Treatment of atherosclerosis
ICK peptides [7,44,64]	-Use as Bioinsecticide -Neuroprotective effect	Analgesic drugs
Hyaluronidase [6,9,78]	-Adjuvant for drugs absorption -Resorption of fluids -Diagnostic allergy test -Delivery of chemotherapy	Contraceptive method
Serpin [9,78]	-Inflammatory modulation -Agricultural pest regulators	Antitumoral drugs
TCTP [9,10,78]	-Antiparasitic effect -Dental restoration -Drug delivery	-N.A

N.A: not applied.
